# Clinical Prediction of Deeply Infiltrating Endometriosis before Surgery: Is It Feasible? A Review of the Literature

**DOI:** 10.1155/2013/564153

**Published:** 2013-09-05

**Authors:** Márcia Mendonça Carneiro, Ivone Dirk de Sousa Filogônio, Luciana Maria Pyramo Costa, Ivete de Ávila, Márcia Cristina França Ferreira

**Affiliations:** ^1^Department of Obstetrics and Gynecology, Universidade Federal de Minas Gerais, Av. Prof. Alfredo Balena, 190, 30130-100 Belo Horizonte, MG, Brazil; ^2^Biocor Hospital Belo Horizonte, R. da Paisagem, 280, Vila da Serra, 34000-000 Belo Horizonte, MG, Brazil

## Abstract

*Background*. Endometriosis is a chronic benign gynecologic disease that can cause pelvic pain and infertility affecting almost 10% of reproductive-age women. Deeply infiltrating endometriosis (DIE) is a specific entity responsible for painful symptoms which are related to the anatomic location of the lesions. Definitive diagnosis requires surgery, and histological confirmation is advisable. The aim of this paper is to review the current literature regarding the possibility of diagnosing DIE accurately before surgery. Despite its low sensitivity and specificity, vaginal examination and evaluation of specific symptoms should not be completely omitted as a basic diagnostic tool in detecting endometriosis and planning further therapeutic interventions. Recently, transvaginal ultrasound (TVUS) has been reported as an excellent tool to diagnose DIE lesions in different locations (rectovaginal septum, retrocervical and paracervical areas, rectum and sigmoid, and vesical wall) with good accuracy. *Conclusion*. There are neither sufficiently sensitive and specific signs and symptoms nor diagnostic tests for the clinical diagnosis of DIE, resulting in a great delay between onset of symptoms and diagnosis. Digital examination, in addition to TVS, may help to gain better understanding of the anatomical extent and dimension of DIE which is of crucial importance in defining the best surgical approach.

## 1. Introduction

Endometriosis is a progressive and benign estrogen-dependent disease defined by the presence of endometrial tissue (glands and stroma) outside the uterine cavity [1]. Diagnosis and treatment of endometriosis are among the most common indications for laparoscopic surgery today as the disease occurs in reproductive-age women and often leads to pelvic pain and/or infertility [2, 3]. Based on the few reliable data, the prevalence of the condition can reasonably be assumed to be around 10%, but it varies with the population being studied [2, 4].

There are no sufficiently sensitive and specific signs and symptoms nor diagnostic tests for the clinical diagnosis of endometriosis. The clinical presentation is variable, with some women experiencing severe symptoms while others remain asymptomatic [5, 6]. As there is a lack of pathognomonic symptoms and no useful noninvasive clinical tests to diagnose symptomatic disease are available, a delay in the diagnosis that averages from five to 11 years is observed [7]. A relationship between an increase in pain intensity and a decrease in quality of life has been reported in these women.

The need for an invasive diagnostic tool, the complex clinical presentation, the multivariated morphology of endometriotic lesions, and lack of well-designed studies with sufficient numbers of patients hamper research and delay diagnosis and appropriate treatment of the disease [8].

In spite of the plethora of studies available, the pathogenesis of endometriosis remains elusive. Apparently, there are three types of endometriosis: superficial endometriosis, ovarian endometrioma and deeply infiltrating endometriosis (DIE). DIE is considered a specific entity which has been arbitrarily defined in histological terms as endometriotic, lesions extending more than 5 mm underneath the peritoneum [9, 10]. DIE is responsible for painful symptoms [11], whose severity is strongly correlated with the depth of the DIE lesions [10–12].

Ultimately, the diagnosis of endometriosis is usually confirmed or refuted by laparoscopy, preferably performed in conjunction with histologic evaluation of excised lesions [3, 6, 13]. Such diagnosis however requires an experienced surgeon as the varied appearance of endometriosis allows less obvious lesions to be overlooked and does not rule out a number of pathological lesions that may mimic the disease. Lack of consistency between laparoscopic and histologic diagnosis of endometriosis is also a problem [1, 3, 14]. In addition, laparoscopy is a costly invasive procedure requiring general anesthesia, and it may be associated with rare but potentially severe complications. Thus, efforts to identify noninvasive diagnostic tests to detect endometriosis cannot be overemphasized [15]. Nonetheless, some authors advocate that a clinical diagnosis of endometriosis alone based on a structured evaluation with detailed history-taking, physical examination and the appropriate use of imaging techniques might be as accurate as laparoscopy [16–19]. 

Transvaginal ultrasound (TVUS) is a noninvasive reliable test that has been used in the diagnosis of endometriosis for a long time. Its low cost, tolerability, and availability worldwide make it a very useful tool for gynecologists. However, the experience of the examiner can exert profound influences on the results and its reproducibility. The method has specific applications and limitations, but it has been increasingly valued as a first-line approach for women with suspected endometriosis [20]. The decision to perform surgery for deep endometriosis is mainly clinical. Ultrasonography and other imaging techniques such as magnetic resonace image (MRI) can be useful tools to have a preoperative estimation of the size and lateral extension of lesions and is extremely important for the surgical planning and approach. It remains unclear, however, to what extent preoperative ultrasonography or MRI should influence the decision to perform surgery, or indeed the type of intervention to undertake for deep endometriosis [16–19]. Endometriosis however continues to impair health-related quality of life and work productivity across countries and ethnicities, and women still experience diagnostic delays in primary care [7, 16].

The aim of this article is to review the current literature regarding the possibility of diagnosing DIE accurately before surgery (laparoscopy or laparotomy). We searched The Cochrane Library (September 2012), and PUBMED (1966 to April 2012) for relevant articles. Combinations of medical subject heading terms including “deeply infiltrating endometriosis and symptoms”, “deeply infiltrating endometriosis and clinical sings” “deeply infiltrating endometriosis and physical examination”, and “deeply infiltrating endometriosis and ultrasound”, “deeply infiltrating endometriosis and magnetic resonance imaging” were used. No language restriction was applied and all pertinent articles were retrieved. In addition to that, review articles and Guidelines from the European Society of Human Reproduction and Embryology (ESHRE) and the American Society for Reproductive Medicine (ASRM) as well as books were also consulted.

## 2. Can Clinical History Accurately Predict DIE?

Endometriosis is commonly found in the pelvis affecting the ovaries, the pouch of Douglas, and the uterosacral ligaments, but it can also be found in the colon, urinary tract, and even in the lungs; lesions have been described [2, 21]. The most frequent symptoms of pelvic endometriosis are dysmenorrhea, dyspareunia, chronic pelvic pain (CPP), and infertility [6, 7]. These symptoms however appear to show poor correlation with the stage or anatomical location of endometriosis [22]. Apparently, the symptoms of endometriosis are related to the number and/or location of endometrial implants and the number and/or location of adhesions [23].

Porpora et al. (1999) [24] used a 10-point visual analog scale to evaluate the severity of dysmenorrhea, CPP, and deep dyspareunia preoperatively in 90 consecutive women. Chronic pelvic pain predicted both DIE and ovarian endometriomas with adnexal adhesions. Deep dyspareunia predicted simultaneously deep endometriosis and an ovarian endometrioma with periovarian adhesions. Another study, however, involving a total of 469 women encountered no clear-cut association between stage, site, or morphological characteristics of pelvic endometriosis and pain [25]. 

Fauconnier et al. (2002) [12] retrospectivelly studied 255 women so as to see whether specific types of pelvic pain (dysmenorrhea, dyspareunia, dyschezia, gastrointestinal symptoms, and noncyclical pelvic pain) were correlated with the anatomic locations of DIE. Apparently, the different types of pelvic pain were associated with specific locations of DIE. Deep dyspareunia was correlated with involvement of the uterosacral ligament, painful defecation with the vagina, noncyclic pelvic pain with the bowel, lower urinary tract symptoms with the bladder, and GI symptoms with the bowel and the vagina. Severe dysmenorrhea was not correlated with any DIE location, but was correlated with adhesions in the Douglas pouch.

The relationship between the severity of dysmenorrhea in women with posterior DIE and indicators of the extent of their disease was evaluated by Chapron et al. (2003) [26]. The presence of a rectal or vaginal infiltration by the posterior DIE and extensiveness of adnexal adhesion were the only factors that remained related to severity of dysmenorrhea. Vercellini et al. (2007) [22], on the other hand, studied 1054 consecutive women with endometriosis undergoing first-line conservative or definitive surgery. The association between endometriosis stage and severity of pelvic symptoms was marginal and inconsistent and could be demonstrated only with a major increase in study power.

The relationship between anatomic locations and diameter of endometriotic lesions and severity of perimenstrual dyschezia as a possible location-indicating pain symptom for posterior DIE was also evaluated [27]. A significant correlation between dyschezia and posterior DIE was identified. A positive correlation occurred between severity of dyschezia and lesion diameter and rectovaginal endometriosis but not with anterior rectal wall involvement.

A diagnostic model based on symptoms and history as assessed by a standardized questionnaire to predict posterior DIE was used by Chapron et al. (2005) [16] in 134 women with chronic pelvic pain symptoms. Painful defecation during menses, severe dyspareunia (visual analog scale ≥ 8), pain other than noncyclic, and previous surgery for endometriosis were independent predictors for posterior DIE and thus could be used to help screen and counsel women before surgery. Chopin et al. (2006) [28] showed after multiple regression analysis, that rectal infiltration and the revised American Fertility Society score of implants were the only factors that remained related to dysmenorrhea severity. Chêne et al. (2008) [29], on the other hand, failed to find a relationship between severity of symptoms, quality of life, and the extent of endometriotic lesions at surgery.

Ballard et al. (2010) [30] applied a preoperative questionnaire to 185 women before laparoscopy in order to evaluate whether there were specific pain dimensions that could be useful in the diagnosis of endometriosis. Women with endometriosis were more likely to report their pain as throbbing and more likely to experience dyschezia in comparison to women with an apparent normal pelvis. Chapron et al. (2012) [31] prospectively performed preoperative assessment of type and severity of pain symptoms (VAS) in 300 consecutive women and compared it with the peroperative findings of endometriomas and associated DIE. They concluded that in the case of endometrioma, severe pelvic pain was significantly associated with DIE.

In conclusion, a comprehensive clinical history is useful to identify patients at risk for endometriosis, although establishing the diagnosis of the disease based solely on the risk factors can be misleading as a large group of women with endometriosis remain completely asymptomatic. Defining women at risk could help the identification of those who would benefit from referral for diagnostic laparoscopy. Others, however, found that clinical history and symptoms reports cannot be reliably used for triage of women with chronic pelvic pain. These women should thus be referred to a specialized center for thorough assessment [30, 32]. The early diagnosis of endometriosis would allow the use of effective medical and surgical treatments to control symptoms and improve the long term outcome for patients as well as reduce costs [33]. 

## 3. Can Clinical Examination Accurately Predict DIE?

As the pelvic exam is frequently normal in women with endometriosis, the value of a standard pelvic examination in the diagnosis of endometriosis has been debated in many studies with different conclusions. As far as we know, not many studies have examined the predictive ability of the pelvic exam to diagnose endometriosis. Some published work suggests that pelvic tenderness, a fixed retroverted uterus, tender uterosacral ligaments or enlarged ovaries identified during a standard pelvic exam are suggestive of endometriosis [3, 18, 32]. Performing the clinical examination during menstruation apparently may reliably identify deep endometriosis, cystic ovarian endometriosis, or cul-de-sac adhesions [34]. 

In women with infertility or severe dysmenorrhea, uterosacral nodularity may be highly predictive of endometriosis [18, 19]. The accuracy of bimanual pelvic examination has also been compared to transvaginal sonography and has been shown to be equally accurate, but when the ovaries and the uterus were involved, ultrasound performed better [17]. Eskenazi et al. (2001) [32], however, have found that a positive pelvic exam (uterosacral ligament scarring, nodularity, or pain; nodularity or pain in the pouch of Douglas; vaginal endometriotic lesions; painful or fixed adnexal masses; and fixed uterus or pain on movement of uterus) had a 76% sensitivity and 74% specificity.

The results of routine clinical pelvic examination can vary significantly with location of DIE and thus may not be sufficient for the diagnosis and establishing the location of DIE as the higher the lesion, the poorer the physical examination [35]. The sensitivity and specificity of the digital vaginal examination were also low in cross-sectional study with 104 women with suspected endometriosis [36]. Others suggest that the pelvic exam alone is not capable of diagnosing DIE of the ovaries, the bladder, or the rectum and it should be combined with transvaginal ultrasound in order to enhance diagnostic accuracy [37, 38].

Despite its low accuracy, the pelvic examination remains an important step in the initial assessment of DIE as it allows a better understanding of disease extent which is vital for planning surgery and other therapeutic interventions.

Table 1 summarizes the current literature available on the topic.

## 4. Can Transvaginal Ultrasound (TVUS) Accurately Predict DIE?

Deep pelvic endometriosis may involve the uterosacral ligaments, cul-de-sac of Douglas, vagina, rectum, and occasionally the bladder. As evaluation by physical examination is difficult, imaging techniques are needed to evaluate the location and extent of endometriosis. High-resolution transvaginal ultrasonography and, in selected cases, magnetic resonance imaging (MRI) improve the diagnosis of retroperitoneal pelvic endometriosis as well as the identification of lesions that involve pelvic organs [36–38, 40].

Several studies [36–38, 40, 41] point out that TVUS should be the imaging technique of choice to detect the presence of DIE in the intestines (rectum) or rectovaginal septum. MRI should be reserved for equivocal ultrasound results in cases of rectovaginal or bladder endometriosis [3, 14, 20].

Ultrasound has been used in the diagnosis of endometriosis for years although the method has specific applications and limitations. The experience of the examiner can exert profound influences on the results and its reproducibility. In addition, superficial peritoneal endometriotic foci cannot be seen on ultrasound scans nor in other imaging techniques [40]. A systematic review of various studies has shown that TVUS is an effective tool to both confirm and exclude a diagnosis of endometrioma, with moderate accuracy [41].

Ultrasound has only recently been considered as a suitable tool for the diagnosis of DIE. Several studies provide enough evidence that transvaginal ultrasound is not only useful but also should be the first strategic tool for preoperative mapping of lesions and surgical planning [36–38, 42].

Although TVUS is widely available, a few pitfalls can exert profound influences in the performance of the scan. In addition to the usual gynecological scan, the examiner should visualize the bladder wall, the pouch of Douglas, the vaginal wall and the rectovaginal septum, the rectosigmoid, the retrocervical (uterosacral ligaments and torus uterinus), and paracervical areas (ureteral involvement). DIE can be identified as hypoechoic, sometimes poorly delimited areas, roughly round, infiltrating the organ wall or location and may occasionally contain hyperechoic foci [43]. When the transductor is pressed against the endometriotic focus, patients can complain of deep pain [44].

**Figure 1:**
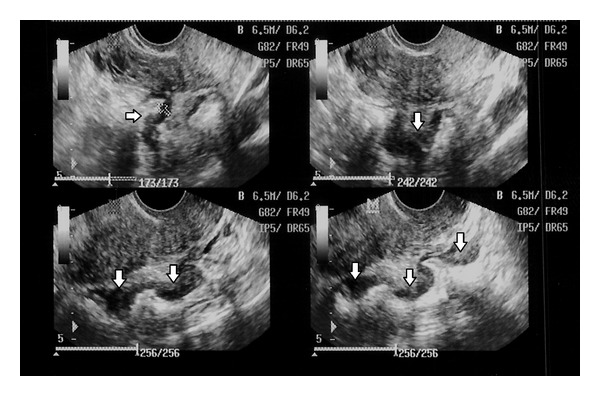
Deeply infiltrative endometriosis lesion of the bowel on ultrasound.

Studies have evaluated the need of specific preparation for TVUS scanning, such as rectal aquous contrast, bowel preparations with laxatives, and vaginal injection of gel. It is not conclusive whether those techniques enhance the performance of the test or should be routinely used [40]. Hudelist et al. (2011) analyzed the diagnostic value of transvaginal sonography (TVS) for noninvasive, presurgical detection of bowel endometriosis and concluded that TVUS with or without the use of prior bowel preparation is an accurate test for noninvasive, presurgical detection of deep infiltrating endometriosis of the rectosigmoid [38] (Figure 1).

**Figure 2:**
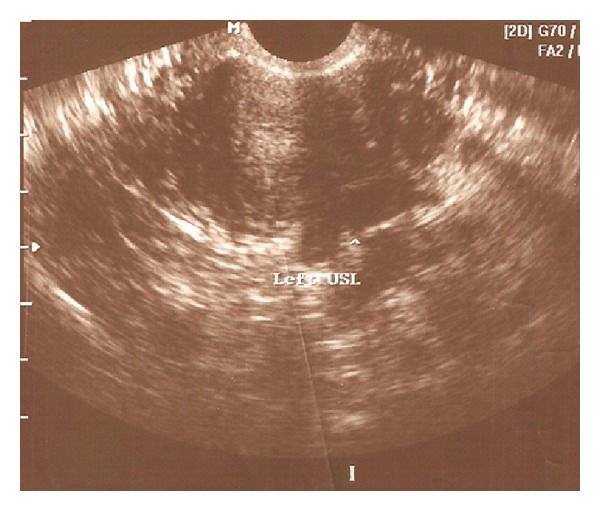
Deeply infiltrating endometriotic lesion of the right uterosacral ligament (arrow) at TVUS. Transversal view of the uterus, at the level of the upper third of the cervix.

TVUS is an accurate method, although sensibility and specificity can vary with the location of the lesion and technique used. Reported sensitivity can vary from a maximum of 98% for intestinal lesions to a minimum of 25% for vaginal lesions. Uterosacral ligaments nodules (Figure 2) are diagnosed with TVS in up to 78% and adequately excluded in up to 88% of the cases [36, 45–48]. 

Intestinal evaluation is extremely important for the surgical planning and approach, since the number of lesions and the depth of invasion influence the composition of the surgical team, the equipment used, and the technique choice. Bowel involvement is frequently multifocal, and the most commonly affected areas are the rectosigmoid colon, the appendix, the cecum, and the distal ileum [49]. Although rectal endoscopic sonographic approach is the most precise for the evaluation of the involvement of intestinal layers, such identification is also possible with TVS. It has been shown that lesions that affect more than 40% of the bowel circumference reach beyond the inner muscular layer [36, 37]. However, the value of digestive layer involvement for choosing the surgical technique has been challenged [50]. 

Vesical and ureteral involvements also influence surgical treatment of endometriosis. In the presence of a paracervical lesion on TVUS, one should suspect of ureteral involvement. Since ureteral obstruction by DIE can lead to hydronephrosis, eventually evolving to insidious renal failure, a urinary tract ultrasound scan may be judicious, followed by specific tests for renal function.

Recently, the use of three-dimensional TVUS (3D TVUS) for the diagnosis of DIE was reported, with good results for vaginal lesions. However, analysis of sensitivity and specificity of 3D TVUS for the diagnosis of DIE in specific sites suggests no striking improvement in comparison to two-dimensional TVUS [51, 52]. 

Being a noninvasive and readily available test in most centers, TVUS is considered a basic step in the evaluation of patients with endometriosis, following and allied to the bimanual pelvic examination. Hudelist et al. (2009) [37] evaluated 155 women with symptoms suggestive of endometriosis in order to compare the diagnostic performance of the pelvic exam with that of TVUS in the presurgical diagnosis of DIE. They concluded that TVUS, performed by skilled staff, clearly enhances diagnostic accuracy, especially in patients with ovarian endometriomas or DIE of the uterosacral ligaments, bladder, and rectosigmoid, but appears to be equally efficient in cases of DIE of the vagina and pouch of Douglas. TVUS is a reproducible method for assessment of the severity of pelvic endometriosis and shows good agreement with findings on laparoscopy [53].

Other imaging techniques are suitable for the diagnosis of endometriosis, such as MRI, that can show all sites of lesions, but we believe that TVUS should be the initial imaging modality, due to immediate availability in most centers, easy access, low cost, and good accuracy for ovarian and deeply infiltrating endometriosis. Data of various studies, regarding sensitivity, specificity, and accuracy of the method for the different locations of deeply infiltrating endometriosis are shown in Table 2. 

## 5. Conclusion

Despite its low sensitivity and specificity, vaginal examination and evaluation of specific symptoms should not be completely omitted as a basic diagnostic tool in detecting endometriosis and planning further therapeutic interventions. Digital examination, in addition to TVUS, may help to gain a better understanding of the anatomical extent and dimension of DIE which is of crucial importance in defining the best surgical approach since extensive bowel involvement warrants an interdisciplinary approach and referral to a tertiary center. TVUS, on the other hand, is highly operator dependent, and good diagnostic results may only be achieved by extensively trained and experienced medical team.

Better diagnostic tools should be continuously sought for, as the approach showed here, although efficient, can sometimes overlook initial pelvic involvement by DIE.

## Figures and Tables

**Table 1:** Relationship between type of chronic pelvic pain (CPP) and deeply infiltrating endometriosis (DIE) lanatomic location.

Author	Type of study (*n*)	Relationship between pain and DIE
Cornillie et al. (1990) [9]	Observational, prospective (*n* 53)	Pelvic pain was strongly associated with deep lesions (>10 mm).

Koninckx et al. (1991) [10]	Observational, prospective (*n* 643)	DIE was strongly associated with pelvic pain, and depth of the lesion was the main factor associated with pain.

Perper et al. (1995) [23]	Double blind observational, prospective (*n* 70)	The intensity of menstrual pain is related to the number of endometrial implants in patients with endometriosis with either pelvic pain or infertility. No diagnosis of DIE.

Vercellini et al. (1996) [39]	Observational, prospective (*n* 244)	Presence of vaginal lesions was associated frequently with severe deep dyspareunia. Stage was not related to pain symptoms.

Porpora et al. (1999) [24]	Observational, prospective (*n* 90)	Deep endometriosis, pelvic adhesions, and ovarian cystic endometriosis were independent predictors of pelvic pain. The severity of dysmenorrhea significantly correlated with the presence and extent of pelvic adhesions. The severity of CPP pain correlated with DIE on the uterosacral ligaments and extent of pelvic adhesions. Deep dyspareunia correlated with DIE on the uterosacral ligaments.

Fauconnier et al. (2002) [11]	Obsevational, retrospective (*n* 225)	The frequency of dyspareunia increased with a uterosacral ligament DIE location. Noncyclic CPP pain was more frquent when DIE involved the bowel. Gastrointestinal symptoms were associated with bowel or vaginal (dyschezia) DIE locations. The frequency of severe dysmenorrhea increased with Douglas pouch adhesions.

Chapron et al. (2003) [26]	Observational, prospective/retrospective (*n* 209)	The presence of a rectal or vaginal infiltration by the posterior DIE and extensiveness of adnexal adhesion were related to dysmenorrhea severity.

Chapron et al. (2005) [16]	Observational, prospective (*n* 134)	The presence of a rectal or vaginal infiltration by the posterior DIE and extensiveness of adnexal adhesion were related to dysmenorrhea severity.

Vercellini et al. (2007) [22]	Observational, prospective (*n* 1054)	A strong association was found between posterior cul-de-sac lesions and dyspareunia. The association between endometriosis stage and severity of pelvic symptoms was marginal and inconsistent and could be demonstrated only with a major increase in study power.

Seracchioli et al. (2008) [27]	Retrospective (*n* 360)	Severity of dyschezia was significantly correlated with posterior DIE. A positive correlation occurred between severity of dyschezia and lesion diameter with rectovaginal endometriosis but not with anterior rectal wall involvement.

**Table 2:** Studies evaluating the accuracy of TVUS for the diagnosis of deeply infiltrating endometriosis in different locations.

Locations studies	Rectovaginal septum			Bowel			Pouch of douglas			Retrocervical area			Uterosacral ligaments			Vagina			Bladder		
	*s*	sp	*a*	*s*	sp	*a*	*s*	sp	*a*	*s*	sp	*a*	*s*	sp	*a*	*s*	sp	*a*	*s*	sp	*a*
Bazot et al., 2003 [45]				95	100	97	82	100	87				75	83	77	25	100	90			
Abrao et al., 2007 [36]				98	100	99				95	98	97									
Menada et al., 2008 [46]	93	90	92	56	92	83															
*RWC	97	100	98	96	100	99															
Piketty et al., 2008 [54]				91	96	NR															
Guerriero et al., 2008 [47]	74	88	NR	67	92	NR							50	94	NR	91	89	NR	100	100	NR
Bazot et al., 2009 [48]	9	99	88	94	100	96							78	67	77	47	95	79			
Goncalves et al., 2010 [55]				97	100	99															

*s*: sensitivity (%); sp: specificity (%); *a*: accuracy (%); NR: not reported.

*RWC: after instillation of rectal water contrast.
